# Academic partnerships for family medicine and primary health care education: a global narrative review 7 years after Astana

**DOI:** 10.3389/fmed.2026.1870375

**Published:** 2026-07-30

**Authors:** Abdul Rehman Zia Zaidi, Raaina Mahevish, Zainudheen Faroog, Racha Kamal Khaled, Maha K. Alyousef, Baraa Alghalyini

**Affiliations:** 1 Department of Family and Community Medicine, College of Medicine, Alfaisal University, Riyadh, Saudi Arabia; 2 School of Kinesiology and Health Science, Faculty of Health, York University, Toronto, ON, Canada; 3 College of Medicine, Alfaisal University, Riyadh, Saudi Arabia; 4 Department of Family and Community Medicine, University of Toronto, Toronto, ON, Canada

**Keywords:** academic collaboration, Astana declaration, curriculum, decolonization, family medicine, North-South partnerships, primary health care, South-South partnerships

## Abstract

The 2018 Declaration of Astana reaffirmed Primary Health Care (PHC) as the foundation for universal health coverage and the health-related Sustainable Development Goals, yet the workforce, curricula, and research infrastructure required to deliver that vision remain unevenly developed across regions. This narrative review, prepared in line with the SANRA reporting standard, examines the global field of academic partnerships for family medicine (FM) and PHC education during the 7 years that followed Astana. We synthesize peer-reviewed and gray literature from 2010 to April 2026 across PubMed, Semantic Scholar, ERIC, and the principal FM and global health journals, and we map the evidence to the World Health Organization three-component PHC framework and Starfield’s 4Cs of primary care. We propose a five-modality typology, the AP-PHC Typology, that distinguishes bilateral North-South capacity-building partnerships, multi-institutional North-South networks, South-South peer-learning networks, triangular partnerships, and multilateral convening platforms. Each modality is mapped to its distinctive contribution across the 4Cs and to the WHO components of integrated services, multisectoral action, and community empowerment. The evidence shows that South-South networks such as PRIMAFAMED, the Iberoamerican Confederation of Family Medicine, AfriWon Renaissance, and the South Asian primary care research community have produced contextually grounded curricula, faculty development pathways, and emerging research traditions. North-South initiatives, in turn, have made measurable but uneven contributions to faculty capacity, often constrained by short donor cycles and asymmetric authorship, with cultural translation rarely fully achieved. Academia is the engine room of post-Astana implementation, but only when partnerships are governed by explicit ethical principles, equitable authorship norms, and sustained financing aligned to country priorities. We close with a falsifiable proposition: where academic FM partnerships explicitly embed the Besrour ten ethical principles and demonstrate equitable LMIC-led authorship, program sustainability beyond donor cycles is significantly more likely than where these conditions are absent. Testing this claim should anchor the next decade’s research agenda.

## 1 Introduction

In October 2018, all 194 World Health Organization (WHO) member states adopted the Declaration of Astana, recommitting the world to Primary Health Care (PHC) as the cornerstone of a health system capable of delivering universal health coverage (UHC) and the health-related Sustainable Development Goals (1, 2). Five years later, in October 2023, ministers, agencies, and civil society returned to Astana to mark the forty-fifth anniversary of Alma-Ata and the fifth of Astana. The dominant message was sober. The world is off-track on health-related SDG 3, the COVID-19 pandemic eroded gains in essential service coverage, and the health workforce gap projected by WHO for 2030 remains stubbornly large in low-income and middle-income countries (LMICs) (3, 4). The World Bank, the Inter-American Development Bank, and the Pan American Health Organization launched the Alliance for Primary Health Care in the Americas in December 2023, in part because pandemic shocks had exposed structural fragility that pre-dated SARS-CoV-2 (5). The Lancet Global Health Commission on Financing Primary Health Care had already warned in 2022 that public spending on PHC in many LMICs was both insufficient and inequitably distributed (6). The 2024 to 2027 Lancet Global Health Commission on People-Centered Care for UHC and the World Bank-PAHO Lancet Regional Health Americas Commission on PHC and Resilience together signal that the global research community has moved past abstract advocacy and is now grappling with implementation at country scale (7–9).

Because this review moves between three related but distinct terms, we define them at the outset and use them consistently thereafter. Primary Health Care (PHC), as set out in the Declaration of Astana and the WHO-UNICEF Operational Framework, is a whole-of-society approach to health that encompasses three components: integrated health services with an emphasis on primary care and essential public health functions; multisectoral policy and action; and empowered people and communities (1, 3). Primary care is the narrower, first-contact clinical service within that approach, providing continuous, comprehensive, and coordinated care for individuals and families over time (10, 11). Family medicine is the academic and clinical discipline that trains physicians to deliver that primary care and, increasingly, to contribute to the wider Primary Health Care agenda (12, 13). These terms are not interchangeable: family medicine education produces a primary care workforce, and a strong primary care workforce is one of several conditions on which Primary Health Care, as a system-level strategy, depends. Where the literature we review concerns generalist primary care delivered outside formally trained family medicine, or system-level Primary Health Care reform rather than clinical service delivery, we signal the distinction explicitly.

Within this turbulent context, the role of academia has been quietly but decisively reframed. The 2018 WHO-UNICEF vision for PHC in the twenty-first century, the 2020 WHO-UNICEF Operational Framework for Primary Health Care, and the WHO Special Programme on PHC have repeatedly returned to a single point: the workforce, governance, and financing reforms that Astana demands cannot be mounted, and quality cannot be sustained, without strong academic systems capable of producing competent generalist clinicians, training their teachers, generating context-relevant research, and informing policy (1, 2, 14, 15). The Lancet Commission on the Education of Health Professionals for the Twenty-First Century had foreshadowed this argument in 2010, calling for a third generation of reforms anchored in transformative learning and interdependent global education systems (12). Family medicine and primary care have a particular stake in this argument. Barbara Starfield’s demonstration that systems organized around the 4Cs of first-contact access, longitudinality, comprehensiveness, and coordination produce better, more equitable, and more efficient health outcomes has only strengthened since 2005 (10, 11). Yet in many countries the educational infrastructure to produce generalists who can deliver these 4Cs at scale is weak, fragmented, or simply absent.

This is the gap that academic partnerships have attempted to fill. Over the past two decades, departments of family medicine and primary care have built bilateral relationships, regional networks, and multilateral platforms whose stated aims range widely. Some focus on curriculum development and residency design; others concentrate on faculty preparation and research mentorship; still others organize around leadership formation and policy translation. The Besrour Centre at the College of Family Physicians of Canada has connected more than forty countries through annual fora and collaborative working groups (13, 16). The Primary Care and Family Medicine Education (PRIMAFAMED) network has woven together approximately forty institutions across twenty sub-Saharan African countries (17, 18). The Iberoamerican Confederation of Family Medicine (CIMF) has shaped regional standards (19). A generation of young doctors movements, including AfriWon Renaissance, the Iberoamerican Waynakay, the South Asian network, and Polaris in North America, has created peer-learning ecosystems that did not exist a generation ago (20, 21). The Network: Towards Unity for Health (TUFH) has anchored the social accountability movement across continents (22). Read alongside one another, these initiatives represent something more than the sum of their parts. Not all of them place Primary Health Care explicitly within their curricula; many are oriented to primary care and family medicine education more narrowly. Taken together, however, they constitute an emerging, if uneven, global academic infrastructure for primary care and family medicine, and a stronger, better-connected academic base of this kind is one of the conditions on which broader Primary Health Care strengthening depends.

That infrastructure is also contested. Critics within the global health community have documented the harms of asymmetric partnerships, the persistence of extractive research practices, the under-representation of Southern authors as first and senior authors on publications about Southern problems, and the way short-cycle donor financing often produces brittle programs that collapse when external funds end (23–26). Reflexive scholarship by Southern authors, including Carvalho et al.’s (24) analysis of Lusophone postcolonial dynamics in health-systems collaboration, Mencía-Ripley et al.’s (25) account of research metrics in the Dominican Republic, and Kesande et al.’s (27) multi-country qualitative study of Global South voices in international partnerships, has reframed the conversation away from charity and toward justice. The Besrour ten ethical principles, articulated by Godard et al. (16), offer one of the few explicit frameworks for navigating these tensions, even though their uptake remains uneven.

This review responds to a specific gap in the post-Astana literature. Excellent reviews of individual partnerships, of curriculum innovations, and of single regions already exist. There is, however, no recent narrative synthesis that maps the full field of academic partnerships for FM and PHC education to the conceptual scaffolding of the three WHO PHC components and Starfield’s 4Cs, that proposes a coherent typology, that integrates the equity and decolonization critique as a structural rather than ornamental concern, and that frames a falsifiable proposition for the next decade. Our aim is to provide that synthesis. The scope is global, the perspective is intentionally pluralist, and the audience is the academic FM and PHC community, the donors and ministries that finance their work, and the editors and commissions now setting the post-Astana research agenda.

## 2 Methods: narrative review approach

This is a narrative review prepared in line with the Scale for the Assessment of Narrative Review Articles (SANRA), which Baethge et al. (28) developed to provide a parsimonious quality framework for non-systematic syntheses. The choice of narrative over systematic methodology was deliberate. The literature on academic partnerships in PHC is methodologically heterogeneous, ranging from descriptive case studies and consensus statements through mixed-methods evaluations to a small number of quasi-experimental designs; aggregating such varied evidence under a PRISMA-style extraction protocol would impose false comparability on the underlying work. We also wanted to make a conceptual contribution, namely a typology mapped to two pre-specified frameworks, and that kind of contribution requires interpretive judgment rather than counting. A narrative approach was particularly important for capturing material from regional journals such as the African Journal of Primary Health Care and Family Medicine, Education for Primary Care, Ciência and Saúde Coletiva, and the Atención Primaria series, since these venues carry much of the most consequential work on Southern-led initiatives but are not always fully indexed by mainstream bibliographic platforms.

The search strategy combined database queries with hand-searching of journals and citation tracking. Databases included PubMed, Semantic Scholar, Google Scholar, ERIC, and the journal sites of BMJ Global Health, the African Journal of Primary Health Care and Family Medicine, Education for Primary Care, Family Medicine, BMC Medical Education, Medical Teacher, the Canadian Family Physician (in particular the Besrour Papers series), the Lancet Global Health and the new Lancet Primary Care, Annals of Global Health, Academic Medicine, and Annals of Family Medicine. Search terms combined controlled vocabulary and free text around primary health care, family medicine, general practice, academic partnership, North-South cooperation, South-South cooperation, twinning, capacity building, curriculum, residency, faculty development, decolonization, equitable authorship, and the relevant policy artifacts (Astana Declaration, WHO Operational Framework, Lancet Commissions). The temporal scope was 2010 to April 2026, with the deliberate inclusion of seminal earlier works such as Starfield et al.’s (10) Milbank synthesis, Macinko et al.’s (11) health systems analysis, and the 2010 Lancet Commission on health professionals’ education (12). The 2010 cut point reflects the publication of the Frenk Commission, which marked a generational shift in how the field talked about academic responsibility for global health.

Inclusion was guided by relevance rather than design hierarchy. We included peer-reviewed empirical studies, narrative and scoping reviews, consensus statements, official policy documents from WHO, UNICEF, the World Bank, PAHO, and WONCA, and selected commentaries that have been highly cited or that articulate widely used frameworks. We excluded sources that did not address academic partnerships directly, sources that addressed only undifferentiated international medical education without a PHC focus, and sources whose claims could not be verified against an accessible primary record. We have not included material from any partnership in which the authors’ affiliated institutions are direct participants, in order to avoid actual or perceived conflicts of interest and to keep the review globally balanced.

Because this is a narrative rather than a systematic review, screening and selection were purposive rather than exhaustive, and we describe the process here in the interest of transparency. One author (ARZZ) conducted the initial searches and assembled a working corpus, reading titles and abstracts to remove items that were clearly out of scope and retaining any record that addressed an academic partnership with an explicit FM or PHC educational dimension. Full texts of retained records were then read by at least one author, and partnership examples were selected for inclusion in the synthesis on three grounds: documentation quality, meaning that the partnership had been described in a citable peer-reviewed or official source rather than only in promotional material; illustrative value, meaning that it exemplified a modality, a mechanism, or a tension that recurred across the wider literature; and geographic and structural diversity, so that the final set did not over-represent the well-funded North-South collaborations that dominate the indexed record. Synthesis proceeded thematically rather than by quantitative aggregation. We grouped sources by partnership structure and by the conceptual frameworks they engaged, mapped recurring claims against Starfield’s 4Cs and the three WHO PHC components, and used points of disagreement between sources to surface the tensions discussed in Sections 8 through 10. Where multiple sources described the same partnership, we privileged the most recent peer-reviewed account and cross-checked factual claims against any official program documentation. This process is interpretive by design, and a different team might reasonably have selected different exemplars; what we claim is not completeness but a defensible and transparent basis for the patterns we describe.

Reflexivity matters in a review of this kind. We approached the literature with the prior conviction that family medicine and primary care are necessary, though not sufficient, conditions for equitable health systems, and that ethically governed academic partnerships are a legitimate vehicle for advancing them. We made a deliberate effort to foreground LMIC-led scholarship, particularly from sub-Saharan Africa, Latin America, South Asia, and Southeast Asia, and to read Northern partnership accounts alongside the reflexive Southern critique rather than as the unmarked default. We acknowledge the persistent risk that even a well-intentioned synthesis can reproduce hierarchies of citation. We have tried to mitigate but cannot claim to have eliminated this risk. Where the evidence is thin, contested, or shaped by donor framing, we say so.

## 3 The primary health care imperative and academic responsibility

Astana 2018 should be read as a corrective rather than a celebration. The declaration came 40 years after Alma-Ata, in a global context where the original PHC vision had been narrowed in many quarters to selective primary care, vertical disease programs, and underfunded clinic-based services (1). Astana reasserted three components: integrated services across the life course delivered through primary care and essential public health functions; multisectoral policy and action on the determinants of health; and empowered people and communities (1, 2). The 2020 WHO-UNICEF Operational Framework translated those components into fourteen levers, four core (political commitment, governance, funding, engagement) and ten operational (models of care, workforce, medicines, infrastructure, digital, quality, primary care research, monitoring, multisectoral action, and community participation) (14, 15). Read in tandem with Starfield’s 4Cs, the framework offers the most coherent contemporary account of what a high-functioning PHC system actually does (10, 11).

The financing picture has been bracing. Hanson et al. (6), on behalf of the Lancet Global Health Commission on Financing Primary Health Care, found in 2022 that PHC remains chronically underfunded relative to its potential health and equity returns, that out-of-pocket spending continues to push tens of millions of households into poverty annually, and that public financing arrangements in many LMICs are not aligned with the integrated, person-centered care that Astana envisaged. The 2024 Lancet Commission on Investing in Health 3.0, often referenced as the Global Health 2050 report, set the audacious target of halving the probability of premature death by mid-century, an outcome the commission argued is feasible only with strengthened PHC at the foundation of national health systems (29). The Lancet Global Health Commission on People-Centered Care for UHC, convened from October 2024 to September 2027, is now explicitly grappling with the operational meaning of person-centeredness, having selected its thirty-four commissioners through an open global call that signals a shift toward more transparent governance of large policy-research enterprises (7, 8). The PAHO-World Bank-Lancet Regional Health Americas Commission on PHC and Resilience, launched in January 2024, has framed PHC as a cornerstone of pandemic and climate resilience for the region, with its first major report appearing in late 2025 (9).

Academia sits at the intersection of all of these reform agendas. The workforce element of the WHO operational framework cannot be delivered without medical schools, residency programs, and continuing professional development systems that produce, retain, and develop generalist clinicians and their interprofessional teams. Research capacity is no different. Real implementation of the research lever requires national and regional research infrastructure that allows LMIC institutions to lead, not merely to participate in, multi-country studies. The quality lever, in turn, depends on educational systems that equip clinicians and managers to use measurement, feedback, and improvement methods, while the community engagement lever depends on graduates who understand themselves as accountable to the communities they serve rather than to professional guilds. Frenk et al. (12) framing of a third generation of health professionals’ education, anchored in transformative learning, instructional reforms, and global interdependence, remains the most influential articulation of this academic responsibility. Boelen’s AMEE Guide 109 on socially accountable medical schools translated those ideas into operational guidance for schools willing to be assessed by the health needs of the populations they serve (30). Building on social accountability, The Network: Towards Unity for Health has developed an international community of practice that now includes designated Centers of Excellence in India, Brazil, the United States, and elsewhere (22).

Read against the 2030 Agenda, academic partnerships for PHC are best understood not as a peripheral collaboration agenda but as core infrastructure for several Sustainable Development Goals. The most direct connection is to SDG 3 (good health and well-being), where target 3.c on increasing health workforce density and retention in LMICs is unattainable without sustained academic capacity, and target 3.8 on universal health coverage hinges on the kind of generalist, equity-oriented, primary-care-centric workforce that academic FM partnerships are designed to produce. SDG 4 on quality education is operationalized through every faculty development pathway, every curriculum partnership, and every accreditation collaboration described in this review. SDG 10 on reduced inequalities is engaged through the explicit equity orientation of the Besrour ethical principles, the bidirectional IMG framing of Akey et al. (31), and the decolonization scholarship of Kesande et al. (27), each of which insists that partnerships should narrow rather than widen structural asymmetries. The most explicit alignment, however, is with SDG 17. Academic partnerships are partnerships in the SDG sense. Target 17.6 on enhanced cooperation in science, technology, and innovation, target 17.9 on capacity-building support, and target 17.16 on multi-stakeholder partnerships for sustainable development together provide the formal vocabulary for what the field has been doing under different names for decades. The implication is consequential. If academic FM partnerships are SDG implementation infrastructure rather than discretionary scholarly cooperation, then they should be planned, financed, evaluated, and reported with the same seriousness applied to any other SDG implementation lever, and unequal or extractive partnerships should be recognized for what they are: failures of SDG 17, not successes of SDG 3.

If Astana sets the destination, academic systems are the route. The post-Astana decade will be judged in part by whether the global academic FM community has used partnerships to accelerate that journey, or whether partnerships have substituted activity for transformation. SDG 3 commitments to health and well-being are inseparable from SDG 17 commitments to partnerships, but the latter are only as good as their governance. Unequal partnerships do not advance SDG 17. They parody it. This review argues, on the evidence, that academic family medicine has begun to take this distinction seriously but has not yet institutionalized it.

## 4 A typology of academic partnerships for primary health care

**Figure 1:**
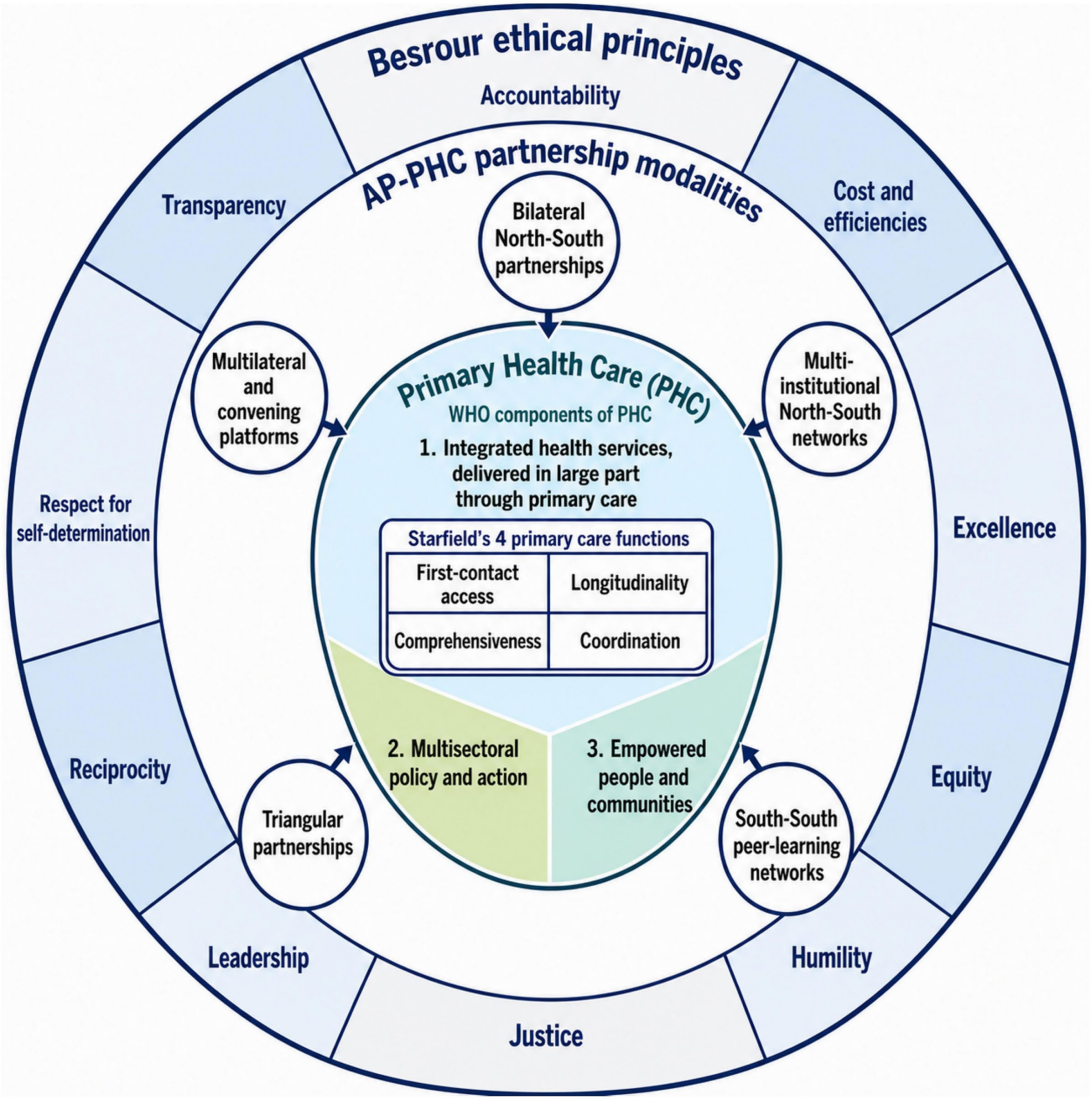
Conceptual schema linking Primary Health Care functions, WHO Primary Health Care components, academic partnership modalities, and ethical principles. The figure organizes Primary Health Care (PHC) as the overarching frame within which the other elements are nested. The innermost layer is the WHO description of PHC and its three interrelated components: integrated health services (delivered in large part through primary care), multisectoral policy and action, and empowered people and communities. Within the integrated-services component, Starfield’s four primary care functions specify what first-contact clinical care contributes: first-contact access, longitudinality, comprehensiveness, and coordination. The next layer shows the five academic partnership modalities of the proposed AP-PHC Typology: bilateral North-South partnerships, multi-institutional North-South networks, South-South peer-learning networks, triangular partnerships, and multilateral platforms. The outer ring binds the ten Besrour ethical principles that should govern all such partnerships.

The literature on academic partnerships in FM and PHC includes valuable descriptions of individual collaborations, but it has lacked an integrative typology that distinguishes structurally different forms of engagement. We propose the AP-PHC Typology (Academic Partnerships for Primary Health Care), a five-modality framework derived inductively from the case literature and mapped deductively to Starfield’s 4Cs and the three WHO PHC components (Table 1; Figure 1). The typology is not hierarchical. Each modality has distinctive strengths, limits, and ethical risks, and well-designed national strategies typically combine several.

**Table 1:** The AP-PHC typology of academic partnerships for primary health care.

Modality	Definition	Exemplar	Starfield 4Cs	WHO 3 components	Strengths	Limitations
1. Bilateral North-South capacity-building	One Northern and one Southern institution paired for FM program design and faculty development	Toronto-Chile (CIPPHC); McGill-Santa Marcelina; Melbourne-CMC Vellore	First-contact access; comprehensiveness	Integrated services	Depth; relational continuity; tailored mentoring	Personnel-dependent; donor-cycle vulnerability; authorship asymmetry risk
2. Multi-institutional North-South networks	Multiple Northern anchors and Southern partners under a networked structure	Besrour Centre; WONCA-WHO joint initiatives	Longitudinality; coordination	Integrated services; multisectoral action	Scale; institutional memory; cross-country comparability	Governance complexity; risk of Northern administrative dominance
3. South-South peer-learning networks	Networks among LMIC institutions, often with light Northern facilitation	PRIMAFAMED; CIMF; AfriWon Renaissance; WONCA South Asia	Comprehensiveness; coordination	Multisectoral action; community empowerment	Contextual fit; cultural authority; emerging research traditions	Financing fragility; uneven research infrastructure; under-representation in indexed literature
4. Triangular partnerships	Southern hub plus Northern technical partner plus Southern emerging program	South African twinning project (Belgian/Canadian support); Mais Medicos (Cuba-Brazil-PAHO)	Coordination; first-contact access	Integrated services; multisectoral action	Disrupts North-South hierarchy; leverages Southern expertise	Political fragility; complex accountability; weak evaluation literature
5. Multilateral and convening platforms	Global agencies, professional bodies, commissions providing standards and convening	WHO Special Programme on PHC; WONCA; Lancet Commissions; The Network: TUFH; AFREhealth	Longitudinality at system level	All three components	Standard-setting authority; cross-regional learning; legitimacy	Distance from frontline implementation; risk of declarative rather than operational outputs

The typology was constructed in three steps. First, reading across the partnership accounts in our corpus, we coded each described collaboration for a small number of structural attributes: the number and type of participating institutions, the direction of resource and knowledge flow, the locus of governance and agenda-setting, and the principal educational function. Second, we grouped collaborations that shared these attributes, and found that they clustered into five recurring configurations rather than lying on a single continuum, which is why the typology is organized by kind rather than by degree. Third, we mapped each emergent configuration deductively onto two established frameworks, Starfield’s 4Cs and the three WHO PHC components, to test whether the distinctions were not merely descriptive but had implications for the primary care functions and system goals each modality is best placed to advance. The resulting categories are analytic ideal types, and real partnerships rarely sit cleanly within one. A bilateral relationship may mature into a multi-institutional network, a South-South network may incorporate a Northern technical partner and so take on a triangular character, and large programmes frequently operate across several modalities at once. The typology is therefore intended to describe dominant structural logics at a given moment rather than to assign fixed and permanent labels, and its purpose is to support comparison, design, and evaluation rather than to police boundaries. Its principal limitation follows from this: because the categories are derived from a purposively assembled and English-dominated literature, they are best understood as a provisional scaffold open to revision as the evidence base widens, particularly as more South-led accounts enter the indexed record.

The first and most familiar form is the bilateral North-South capacity-building partnership, in which a single Northern institution, often a department of family medicine, pairs with a single Southern institution to support the design, accreditation, and faculty development of a postgraduate FM program. Among the more visible examples is the Toronto International Program to Strengthen Family Medicine and Primary Care, which has provided immersive faculty development for visiting scholars from across Africa, Asia, and Latin America (13, 21). McGill University’s long-running partnership with Santa Marcelina in São Paulo has focused on faculty development and research capacity (16), and the University of Melbourne’s institutional engagement with Christian Medical College Vellore has produced more than 120 jointly authored research publications across primary care and related domains since 2012 (32). Bilateral partnerships are well suited to building first-contact access and comprehensiveness because they tend to produce graduates and faculty oriented to undifferentiated generalist care, mapping most clearly to the WHO component of integrated services. Their limits are familiar to anyone who has worked in this space: vulnerability to personnel turnover at either end, dependence on intermittent project funding, and the persistent risk of asymmetric authorship and credit allocation.

A second modality, multi-institutional North-South networks, brings several Northern and Southern institutions into a deliberately networked structure with distributed governance, typically anchored administratively in the North. The Besrour Centre at the College of Family Physicians of Canada is the clearest exemplar. Its member network spans more than forty countries, its working groups cover undergraduate education, leadership, research, and quality improvement, and its annual Besrour Forum has run since the early 2010s (13, 16, 33). The 2018 letter of intent between the College of Family Physicians of Canada and the West African College of Physicians, signed at the seventh Forum, illustrates how the modality scales beyond one-to-one ties (33). Networked structures of this kind expand longitudinality and coordination by creating durable institutional memory and a multi-country pipeline. They map to integrated services and, when they incorporate patient and community partners into governance, to community empowerment as well.

A third modality, South-South peer-learning networks, is the form most under-represented in mainstream global health discourse and arguably the most generative in the post-Astana decade. PRIMAFAMED is the standard-bearer. Originally seeded by Ghent University in Belgium in 2007, the network is now wholly steered by African institutions and currently spans approximately forty institutions in twenty countries (17, 18, 34). A series of milestone outputs traces its trajectory: the 2016 Nairobi consensus on undergraduate FM training across eighteen sub-Saharan universities; the 2022 Malawi conference on education for PHC; the 2024 mapping of undergraduate teaching across the network; and the 2025 cross-network survey of planetary health continuing professional development across thirty-eight institutions and twenty-four countries (17, 18, 35, 36). These outputs together represent a Southern-led research and curriculum agenda of remarkable depth. Other South-South structures matter too. The Iberoamerican Confederation of Family Medicine, the sixth WONCA region, has produced regional educational standards and shaped the Latin American conversation since 1981 (19, 37). AfriWon Renaissance, working through the AfriWon Research Collaborative, has built peer-mentorship models for early-career family physicians and registrars across western, central, and southern Africa (20, 38, 39). And the South Asian primary care research network, anchored under WONCA South Asia, has begun to articulate a regional research agenda for India, Pakistan, Bangladesh, Nepal, Sri Lanka, and Bhutan (40). South-South networks excel at contextual relevance, comprehensiveness, and community empowerment. Where they typically struggle is in financing fragility and uneven research infrastructure.

A fourth modality, triangular partnership, pairs a Southern hub institution with a Northern technical partner and one or more Southern emerging programs. The cleanest documented example is the Strengthening Developmental Capacity for Family Medicine Training in Africa initiative, in which South African departments of family medicine twinned with emerging programs in countries lacking medical faculties while drawing on Belgian and Canadian technical and financial inputs (34). A more politically charged variant is the Mais Médicos program. Between 2013 and 2018 the Pan American Health Organization brokered the deployment of approximately seventeen thousand Cuban primary care doctors to underserved Brazilian municipalities, with Cuba supplying the workforce, Brazil supplying the policy and reimbursement architecture, and PAHO supplying the multilateral scaffolding (41–43). When ethically governed, triangular partnerships are uniquely positioned to advance coordination across the three WHO PHC components and, just as importantly, to disrupt the implicit hierarchy of bilateral North-South arrangements.

The fifth modality, multilateral and convening platforms, constitutes the global commons of academic PHC. WHO’s Special Programme on Primary Health Care, WONCA’s working parties on education, research, and rural practice, the Lancet Commissions, AFREhealth, and The Network: TUFH together provide standards, evidence syntheses, and platforms for cross-country learning that no single bilateral partnership can supply (14, 22, 29, 44). The Strengthening Primary Health Care Leadership Global Capacity Building Course, launched by WHO Academy in autumn 2024 in collaboration with academic partners, is a contemporary illustration of how multilateral platforms can directly deliver leadership formation aligned to the operational framework (44). The Global Coalition of Countries on PHC, with eighteen member countries by mid-2025, signals that the convening function is moving from declaration to implementation (45). Multilateral platforms are uniquely positioned to advance the multisectoral policy and community empowerment components and to drive longitudinality at the system level.

The typology is intended as a working scaffold, not a closed taxonomy. Most national family medicine systems sit at the intersection of several modalities. The most resilient appear to combine at least three: a strong South-South network membership, one or two carefully governed bilateral relationships, and engagement with multilateral platforms. Table 2 catalogs thirty-seven empirical academic partnerships across the five modalities, and Figure 1 places the typology within the wider conceptual frame that ties Starfield’s primary care functions, the WHO PHC components, the partnership modalities, and the Besrour ethical principles into a single accountability schema.

**Table 2:** Evidence summary of empirical academic family medicine and primary health care partnerships.

#	Partnership	Partners (region)	Type	Educational focus	Duration	Key reported outcomes	Citation
1	Besrour Centre for Global Family Medicine	CFPC + ~ 40 LMIC academic FM departments	N-S networked	UME/PGME/Faculty dev	2012-present	10 ethical principles; global FM atlas; faculty needs assessment	(13, 16, 26, 33, 46, 47)
2	PRIMAFAMED Network	Stellenbosch, Ghent + ~ 40 sub-Saharan African universities	S-S + triangular	UME/PGME/CPD	2007-present	73-article evidence base; 24-country planetary health CPD; ECSA College proposal	(17, 18, 34–36, 67)
3	Toronto International Program (CIPPHC)	U Toronto DFCM + Chilean Ministry of Health	N-S bilateral	CPD/IPE	2010–2012+	Three cohorts evaluated; cross-country research training	(21, 50)
4	Programa Mais Medicos	Brazilian MoH + Cuban MoH + Brazilian universities + PAHO	S-S + triangular	Workforce + UME curriculum reform	2013-present	~17,000 physicians placed; reductions in amenable mortality in high-need municipalities	(41–43)
5	Brazil Family Health Strategy academic linkages	Brazilian universities + Sistema Unico de Saude	National + advisory	UME/PGME/CPD	1994-present	World’s largest CHW-physician PHC model; documented mortality reductions	(41, 43, 68)
6	Besrour COVID-19 vaccine integration study	32 countries	Multilateral	Research/CPD	2022–2024	PC-PH integration positively associated with vaccination coverage in LMICs	(53)
7	Besrour ethical framework consultation	12 LMIC universities + CFPC	N-S networked	Faculty dev	2014–2017	Ten ethical principles + decision tool	(16)
8	Besrour LMIC faculty development needs	12 LMIC universities	N-S networked	Faculty dev	2015	Identified gaps in teaching skills and FM-specialist relationships	(51)
9	Manitoba/McMaster Ethiopia capacity building	Manitoba + McMaster + Addis Ababa	N-S bilateral	PGME/Faculty dev	2010–2018	Sustainable Ethiopian residency program developed	(49, 52)
10	Besrour Global Primary Care Research Capacity-Building	NAPCRG + Ottawa + Stellenbosch + Jos + Auckland + Guyana	Multilateral	Research capacity	2019-present	Five LMIC case studies (Africa, SE Asia, South America)	(33)
11	Proposed ECSA College of Family Medicine	East/Central/Southern Africa + South Africa + WONCA-Africa	S-S + triangular	PGME governance	Proposal 2022	Regional accreditation framework	(65)
12	PRIMAFAMED Planetary Health CPD	38 institutions, 24 sub-Saharan African countries	S-S networked	CPD	2024–2025	80 respondents; ranked clinical priorities; web-based CPD preferred	(36, 77)
13	PRIMAFAMED Climate-Resilience CPD	8 sub-Saharan African countries	S-S networked	CPD	2024	Six learning needs identified through qualitative analysis	(77)
14	Creighton AGSPP	Creighton + Rwanda + Nepal + Ecuador + Dominican Republic	N-S decolonized	UME/MPH (5 yr)	2017-present	Three-component decolonized framework; MEL platform	(59)
15	California IMG bidirectional pathway	California AMCs + LMIC institutions	N-S	UME/PGME	2020s-present	Identified visa and policy barriers; Special Permits pathway	(31)
16	Pediatric Global Health Leadership Network	36 US AMCs + LMIC partners	N-S	PGME/Faculty dev	2015-present	Priority-setting and educational implementation frameworks	(56, 57)
17	Lusophone Africa health researcher mobility	Portugal + Brazil + Mozambique + Angola + Cape Verde + Guinea-Bissau	N-S Lusophone	Research capacity	2015–2018	Postcolonial reflection on capacity-building	(24)
18	Universidad Iberoamericana COVID-19 response	Dominican Republic + multilateral	South-led	Research/policy	2020-present	Decolonial science diplomacy case	(25)
19	African fellows-UC Berkeley	Uganda/Cameroon/SA + UC Berkeley	N-S, South-led voice	MPH/Research	2022–2024	Four equity strategies articulated by Southern fellows	(27)
20	Lancet Global Health Commission on Financing PHC	22 commissioners, 15 countries	Multilateral	Policy/research synthesis	2020–2022	Three-volume policy framework	(6)
21	WHO/U Toronto/Aga Khan PHC Leadership initiative	WHO Academy + U Toronto + Aga Khan + global	Multilateral	Leadership development	2024-present	First global PHC leadership program	(44)
22	McLellan team-based PC paper consortium	U Toronto + WHO + Athabasca	N + multilateral	Curriculum/IPE	2026	Call for relational transformation pedagogy	(75)
23	Northern Ontario School of Medicine model	Canada + Australia + Philippines	N-N + triangular	UME LIC	2005-present	Typology of LICs; rural retention evidence	(73, 74)
24	Socially Accountable Med School (THEnet)	13 + countries	Multilateral	UME	2008-present	AMEE Guide 109 framework; ISAT 2.0 instrument	(22, 30, 79)
25	Brazil Mais Medicos Cuban physician mobility	Brazil + Cuba + PAHO	S-S	Workforce	2013–2018	11,400 + Cuban physicians placed	(41, 43)
26	Global Family Medicine Atlas	Wilfrid Laurier + Besrour	N-led, global mapping	Mapping/policy	2018–2023	Country-level FM training data	(26)
27	AfriWon Research Collaborative pilot	Boston U + Botswana + AfriWon	N-S + S-S	PGME research mentorship	2018–2021	Online mentorship pilot; 35% completion rate	(38, 39, 70)
28	Lancet HQSS Commission	30 + countries	Multilateral	Health systems education	2016–2018	Quality-education linkage framework	(80)
29	Besrour narrative inquiry project	16 LMIC partners	N-S networked	Qualitative narrative	2012–2014	17 success themes	(48)
30	Besrour evidentiary basis project	Canada + multiple LMIC	N-S	Research methodology	2013–2015	Methodological challenges in evaluating FM globally	(13)
31	Frenk Lancet Commission on HP Education	20 commissioners, multiple regions	Multilateral	UME/PGME global reform	2008–2010	Transformative + interdependent learning frame	(12)
32	Decolonizing GEM Working Group (GEMA)	100 + members worldwide	Multilateral	EM/PGME	2020-present	Four-priority decolonization framework	(60)
33	Decolonizing photography in academic GH	Johns Hopkins-led; multi-country	N	UME/Faculty dev	2025	Multi-level institutional photographic ethics framework	(61)
34	Reynolds-Bekele 10 questions framework	U Michigan + UGHE Rwanda	N-S	UME/PGME	2023	Equity reflection tool	(58)
35	South Asian primary care research network	India + Pakistan + Bangladesh + Nepal + Sri Lanka + Bhutan + WONCA SAR	S-S	Research capacity	2020-present	Articulated regional research agenda for South Asia	(40, 71)
36	Iberoamerican CIMF education standards	Spain + Latin America (CIMF/WONCA Region)	S-S networked	UME/PGME/CPD	1981-present	Regional educational standards; standardized curricula	(19, 37)
37	PAHO digital transformation for PHC	PAHO + Latin American MoHs + universities	Multilateral	Digital health pedagogy	2022-present	Regional digital PHC strategy and curricular adaptations	(76)

## 5 North-South partnerships: promises and pitfalls

North-South partnerships are the most documented modality, partly because Northern institutions have the resources to publish and partly because their funders demand evaluation. The Besrour Centre, founded by the College of Family Physicians of Canada in 2015 with the philanthropic support of Dr. Sadok Besrour, has connected Canadian family medicine departments with partners in more than forty countries through education, research, and quality improvement working groups (13, 16). Ponka et al. (13) Besrour paper articulated the Centre’s vision of supporting countries to develop their own academic expertise rather than exporting Canadian practice, a stance that has been operationally reinforced by Rouleau et al. (46) analyses and by Arya et al. (37) work on the Centre’s strategic direction (47, 48). The Centre’s collaboration with WONCA, WHO, the World Bank, Global Affairs Canada, and the Conférence Internationale des Doyens de Facultés de Médecine d’Expression Française has positioned it as a node in a much larger ecosystem (16, 33).

Toronto’s contribution to global family medicine has been documented in detail. Woldeyes et al. (49) described the Toronto Addis Ababa Academic Collaboration in Family Medicine, a capacity-strengthening partnership supporting Ethiopia’s first family medicine residency programme through curriculum support, faculty development, leadership training, and mentorship. Godoy-Ruiz et al. (50) paper on a Toronto-led international primary care and family health program offers an unusually candid account of the iterative negotiation between Northern and Southern partners over agenda-setting, governance, and authorship. Redwood-Campbell et al. (51) study of the family medicine faculty development needs of low- and middle-income country partners, and Dyck et al. (52) piece on Manitoba’s contribution, have added further texture. Sodhi et al. (53, 54) work on primary care and public health integration, including a cross-national study protocol and the 2025 PLOS ONE multi-country study of the relationship between that integration and COVID-19 vaccination coverage, illustrates how mature North-South research partnerships can produce cross-country evidence with policy implications. Ponka et al. (33) BMJ Global Health analysis brought the conceptual contribution of the Besrour Centre into the open access global health literature for the first time. The wider role of Primary Health Care in advancing the Sustainable Development Goals has been set out by Chotchoungchatchai et al. (55).

The literature is increasingly honest about asymmetry. Kulesa et al. (56) have examined how United States-based practitioners set priorities and allocate resources in academic global health partnerships, and have separately analyzed the factors that shape how educational programs are implemented within those partnerships (57). Reynolds et al. (58) Academic Medicine paper proposed ten questions that institutions and individuals should ask before engaging in a global health partnership, ranging from “whose agenda?” to “what happens when funding ends?” Beste et al. (59) paper synthesized nine implementation themes across academic global health partnerships, including the chronic mismatch between donor timelines and program maturation and the persistent under-resourcing of Southern coordinating capacity. Muchatuta et al. (60) work has extended this critique into emergency medicine education, with implications for primary care given the boundary work between PHC and emergency services. Akey et al. (31) paper on bidirectional pathways for international medical graduates argues that the assumption that Southern trainees benefit from Northern rotations while the reverse does not occur is empirically wrong and ethically untenable. Harrison et al. (61) paper on photographic ethics in global health teaching is a small but pointed reminder that even the visual culture of partnerships embeds power.

The Besrour ten ethical principles articulated by Godard et al. (16), namely accountability, cost and efficiencies, excellence, equity, humility, justice, leadership, reciprocity, respect for self-determination, and transparency, were developed specifically as a response to this asymmetry critique. They are best read not as a checklist but as a continuous test. A partnership that scores well on excellence but poorly on equity is not a good partnership. One that scores well on reciprocity but poorly on transparency is fragile. The question for the next decade is whether the principles will be embedded in funder requirements, institutional review, and authorship norms, or remain aspirational. We argue, in Section 12, that this question is empirically testable.

## 6 South-South partnerships: the quiet revolution

If North-South partnerships dominate the indexed literature, South-South partnerships are doing much of the substantive work. PRIMAFAMED is the clearest case. Originating in 2007 as a Belgian-supported network seeded by Ghent University across ten universities in eight sub-Saharan African countries, the network has matured into an African-led structure of approximately forty institutions in twenty countries, with explicit South-South-North governance philosophy (17, 18, 34). At the 2016 Nairobi meeting, eighteen institutions documented the state of undergraduate FM teaching across the region and produced a consensus on what a contextually appropriate undergraduate exposure should include (17). The 2022 Malawi conference brought together twenty-nine institutions from eighteen countries under the theme “Education for PHC in Africa” (62). The 2024 mapping by Mash et al. (18) documented the diversity of undergraduate teaching arrangements across institutions ranging from Amoud University in Somaliland (where family physicians contribute extensively to the general curriculum but have no dedicated clerkship) to Moi University in Kenya (with thirty weeks of community-based clinical exposure across years one to five). The 2025 BJGP Open survey of planetary health continuing professional development drew responses from eighty members of the network across thirty-eight institutions and twenty-four countries, demonstrating both the reach of South-South networks and the continuing knowledge gaps the next decade must address (36, 63).

Mash et al. (64) Human Resources for Health paper on the roles and training of primary care doctors across China, India, Brazil, and South Africa remains a widely cited empirical synthesis of South-South family medicine work. Besigye et al. (17) work on Makerere’s family medicine evolution, and Ray et al. (65) analysis of Botswana’s program, illustrate how networked Southern institutions have generated their own evaluation literature rather than waiting for Northern partners to produce it. De Maeseneer and Flinkenflögel’s (34) British Journal of General Practice paper laid the conceptual groundwork for a sub-Saharan family medicine identity that did not need to mirror European or North American antecedents (66). Flinkenflögel et al. (67) Human Resources for Health scoping review remains the most thorough synthesis of FM training in sub-Saharan Africa, documenting variation in program length, governance, and accreditation across more than a dozen countries.

In Latin America, the Iberoamerican Confederation of Family Medicine has played a similar standard-setting role for the Spanish- and Portuguese-speaking world. Its development of regional educational standards, in dialogue with the WONCA Education Working Party, has shaped curriculum governance from Mexico to Argentina (19, 37). The Brazilian Sistema Único de Saúde has produced a remarkable academic linkage with the Family Health Strategy through programs such as the Programa de Educação pelo Trabalho para a Saúde and the Mais Médicos initiative (41–43, 68). Macinko and Harris (68) New England Journal of Medicine paper made the global case for the Brazilian model, documenting the association between Family Health Strategy expansion and reductions in amenable mortality. Hone et al. (41) difference-in-differences analysis across 5,565 Brazilian municipalities found that Mais Médicos was associated with an increase of 15.1 PMM-contracted primary care doctors per 100,000 population and modest reductions in amenable mortality, with effects concentrated in municipalities with high health burden and low pre-existing physician density. Both of these are quasi-experimental methods that estimate a program’s effect by constructing a comparison for what would have happened in its absence, the difference-in-differences design using trends in unexposed areas and the synthetic-control design building a weighted composite of comparison units. Subsequent generalized synthetic control analyses have been more cautious about long-run impact, suggesting that workforce inputs alone do not generate health outcomes without supportive system architecture (42). The lesson is sobering and useful: South-South workforce mobility is necessary but not sufficient.

The Carvalho et al. (24) paper on Lusophone Africa offers a different angle. Their analysis of Portuguese-speaking African capacity building, including engagement between Portugal, Brazil, Mozambique, Angola, Cape Verde, and Guinea-Bissau, reads colonial linguistic legacies as both an asset (shared scholarly language, cultural familiarity) and a liability (replication of colonial hierarchies in research authorship and funding). Mencía-Ripley et al. (25) paper on the Dominican Republic argues that academic partnerships are strengthened when the local perspective drives the agenda, drawing on a case in which a university took a central role in the national COVID-19 response, and frames science diplomacy through a decolonial lens. Tchonang Leuche et al. (69) paper extends this argument into global child health education, calling for more equitable and culturally safe collaborations and warning that francophone scholarship remains under-represented in English-medium indexed databases.

AfriWon Renaissance, the African young doctors movement of WONCA, has built on these foundations through peer mentorship in research, leadership formation, and conference engagement, including its 2024 emergency medicine pre-conference at Aga Khan University in Nairobi (20, 38, 39, 70). The AfriWon Research Collaborative pilot, led by McGuire et al. (38) with Boston University and Botswana faculty support, demonstrated the feasibility of online research mentorship across western, central, and southern Africa, while honestly reporting that only 35 % of mentees completed the program in the formal evaluation (39). The South Asian Federation of Family Physicians and WONCA South Asia have begun to articulate a parallel research and education agenda for the most populous region in the world, where general practice rather than formally trained family medicine still provides most primary care (40, 71).

## 7 Curriculum innovations born of collaboration

Academic partnerships have been a major engine of curriculum innovation in PHC. Several distinct strands have emerged from the post-Astana decade, with two further strands sharpening into focus since the pandemic.

Competency frameworks have been an early and contested terrain. The Canadian CanMEDS-Family Medicine framework, the ACGME family medicine milestones, and the WONCA global standards have all been adapted, contested, and repurposed across many partnerships, with the Iberoamerican Confederation’s regional standards offering one of the most carefully documented cases of regional translation rather than wholesale import (19, 37). A more recent example comes from the University of Cape Town. von Pressentin et al. (72) redesigned the leadership component of the family medicine MMed in dialogue with national and African regional needs, illustrating how competency frameworks can be reshaped by Southern academic leadership rather than imposed from above.

A parallel strand has been the longitudinal integrated clerkship. Worley et al. (73) Medical Education typology gave the global community a shared vocabulary for distinguishing community-based, longitudinal, and rural clerkship designs, drawing evidence from Australia, Canada, the United States, South Africa, and the Philippines. Strasser et al. (74) work on the Northern Ontario School of Medicine has been particularly influential because it documented how distributed, community-engaged training produces graduates more likely to practice generalism in underserved areas. Adaptations of this design across partnerships, including under the PRIMAFAMED umbrella in sub-Saharan Africa, have shown that the longitudinal model can survive and even thrive in resource-constrained settings, though the resources required for genuine community-based longitudinal training are routinely underestimated (18, 34, 73).

Community-oriented primary care belongs to a longer pedagogical lineage but has had a quiet renaissance in the post-Astana decade. The COPC tradition was developed by Sidney and Emily Kark in South Africa in the 1940s and re-imported across the Atlantic in the 1960s. South African, Israeli, and North American partnerships have continued to refine COPC pedagogy, and Mash et al. (64, 66) work on the contribution of family physicians to district health systems shows how COPC principles map onto modern team-based primary care.

Interprofessional education has moved closer to the center of the conversation. Interprofessional education is the pedagogy through which team-based primary care is meant to be produced, and the gap between the two is where recent scholarship has concentrated. McLellan et al. (75) Frontiers in Medicine paper, written within the same Frontiers Research Topic that anchors the call for the present review, makes a sharp argument: team-based primary care without genuine collaboration is the dominant pattern globally, and academic partnerships must therefore focus on relational transformation rather than on structural co-location. Drawing on the WHO Framework for Action on Interprofessional Education and Collaborative Practice and the IPEC Core Competencies, McLellan et al. (75) argue that relational competencies must be intentionally cultivated through pedagogy from the pre-service phase onwards. Their argument is consistent with empirical evidence from Malaysia, the Netherlands, the United Kingdom, and the United States showing that proximity does not produce collaboration: only deliberate education does (75).

Digital and ECHO-style models entered the mainstream during COVID-19 and have stayed there. Sodhi et al. (53) 2025 PLOS ONE paper on the relationship between primary care and public health integration and COVID-19 vaccination across multiple countries demonstrates how digital data-sharing collaborations can yield cross-country evidence at scale. Project ECHO, originally pioneered for hepatitis C in the United States, has spread into family medicine continuing professional development across Africa, South Asia, and Latin America, with the AfriWon Research Collaborative’s online curriculum serving as a peer-learning analogue (38, 39). Barbosa da Silva et al. (76) articulation of the PAHO digital transformation agenda situates these innovations within a regional strategy.

Planetary health curricula are the newer of the post-pandemic strands. Mash et al. (36)and Lokotola et al. (63) BJGP Open survey across thirty-eight institutions and twenty-four countries in sub-Saharan Africa documented that most respondents were not familiar with planetary health, with sixty point 8 % having never attended related education. Their parallel African Journal qualitative study identified the specific learning needs and adaptation strategies that should anchor a coordinated curriculum agenda over the coming decade (77). The AMEE consensus statement on planetary health and education for sustainable healthcare, the Rockefeller Foundation-Lancet Commission on Planetary Health, and the Africa-led work of Lokotola et al. (63) have collectively created the conditions for such an agenda (36, 77, 78).

Social accountability remains the strand that ties these innovations together. Boelen et al. (30) AMEE Guide 109 remains the operational reference for socially accountable medical schools, and The Network: TUFH’s Indicators of the Social Accountability Tool (ISAT 2.0), revised in 2024, gives institutions a structured self-assessment instrument now in use across the Americas, Africa, South Asia, and Southeast Asia (22, 79). Whether to integrate social accountability into accreditation systems is a live policy debate in several countries, and the answer matters because, without such integration, the curricular innovations described here can be quietly defunded when leadership changes.

## 8 Equity, decolonization, and the politics of knowledge

The decolonization debate in global health is no longer at the margins. Kesande et al. (27) Annals of Global Health paper on voices from the Global South, drawing on qualitative interviews with Ugandan and Kenyan partners in international academic collaborations, documents in plain language the recurring patterns of agenda capture, authorship asymmetry, and “parachute research” that have defined the field for decades. Their argument is not that North-South partnerships should end, but that they should be radically reformed. The Besrour ten ethical principles, articulated as a structured framework rather than a slogan, offer a proximate operational vocabulary, and Table 3 maps each principle to the corresponding lever of the WHO Operational Framework with a worked example (16). Reynolds et al. (58) ten questions provide a complementary screening tool.

**Table 3:** The Besrour ten ethical principles mapped to WHO operational framework levers.

Principle	Working definition [Godard et al. (16)]	WHO operational framework lever	Worked example
1. Accountability	Partners are answerable to each other and to the populations they serve	Governance and policy frameworks (core)	Joint accountability frameworks in the Toronto-Chile CIPPHC partnership
2. Cost and efficiencies	Resources are deployed prudently and shared transparently	Funding and allocation of resources (core)	Open budgeting in the Besrour Forum working groups
3. Excellence	All partners commit to high quality in education, research, and service	Models of care; Quality improvement (operational)	PRIMAFAMED standards-of-care work across 24 countries
4. Equity	Partnership benefits accrue equitably across all partners	Engagement with communities and stakeholders (core)	Equitable authorship norms in the AfriWon Research Collaborative
5. Humility	Partners recognize the limits of their own knowledge and learn from each other	Primary care research and learning (operational)	Northern faculty learning COPC pedagogy from South African colleagues
6. Justice	Partnerships actively redress historical and structural inequities	Engagement with communities; Quality (operational)	Decolonization frameworks applied in the Creighton AGSPP
7. Leadership	Partners share leadership and develop emerging leaders from all sides	Health workforce (operational)	WHO/U Toronto/Aga Khan PHC Leadership course (Khalid 2025)
8. Reciprocity	Partnerships create value flowing in all directions	Engagement with communities (core)	Bidirectional IMG pathways (Akey 2026)
9. Respect for self-determination	Southern partners set their own agendas and govern their own institutions	Political commitment and leadership (core)	PRIMAFAMED’s transition from Belgian seeding to African-led governance
10. Transparency	Decisions, finances, and outcomes are openly reported	Monitoring and evaluation; Funding (core)	Open evaluation reporting in the AfriWon online research mentorship pilot

Authorship is the most concrete site of struggle. Mencía-Ripley et al. (25) analysis of decolonizing science diplomacy in the Dominican Republic, Carvalho et al. (24) Lusophone reflection, and Tchonang Leuche et al. (69) francophone West African work converge on the same conclusion: the simple metric of first and senior authorship by LMIC researchers should be a routine component of partnership evaluation. Beste et al. (59) nine implementation themes include explicit attention to equitable authorship metrics. Akey et al. (31) paper on bidirectional IMG pathways pushes the argument into the migration domain, arguing that international medical graduates from LMICs to high-income countries are not simply beneficiaries of training but contributors to high-income health systems whose contributions deserve reciprocal investment. Harrison et al. (61) paper on photographic ethics extends the critique into the visual register, arguing that the iconography of “Northern doctors with Southern children” continues to do real harm and that academic partnerships should adopt explicit visual ethics policies.

Indigenous-led partnerships sit at a particular axis of this debate. The Lancet Americas Commission’s 2025 report explicitly engaged Indigenous representatives, including from the Fund for the Development of Indigenous Peoples of Latin America and the Caribbean (9). Canadian and Australian academic family medicine programs have begun to engage seriously with Indigenous self-determination in curriculum design, although the literature remains thin and often uneven in its handling of community-led versus university-led framings.

Linguistic decolonization is another underdeveloped axis. The dominance of English in indexed global health and FM literature systematically disadvantages francophone, Lusophone, Hispanophone, Arabophone, and Asian-language scholars. The continued vitality of the African Journal of Primary Health Care and Family Medicine, Atención Primaria, the Brazilian Ciência and Saúde Coletiva, and the Journal of Family Medicine and Primary Care from India suggests that multi-language scholarship is alive but under-cited (40, 68, 71). Future bibliometric work on academic partnerships should explicitly document language of publication and impact across language communities.

## 9 Workforce and population health impact

The preceding sections described what partnerships build, namely curricula, faculty, and networks; the harder question, and the one on which the case for academic partnership ultimately rests, is whether these contributions translate into measurable effects on populations. By impact we mean effects on population health outcomes, cost, equity, and access, the dimensions on which primary care has historically been judged. The strongest evidence base for primary care impact remains the foundational work of Starfield et al. (10), whose 2005 Milbank Quarterly paper systematized two decades of cross-national comparisons showing that systems with stronger primary care orientation produce better population health outcomes, lower costs, greater equity, and higher patient satisfaction. Macinko et al. (11) Health Services Research paper extended that argument across OECD countries. The evidence has held up across replications and methodological refinements.

The post-Astana question is whether academic partnerships have demonstrably contributed to these outcomes. The honest answer is that the attribution gap is substantial. We can document with reasonable confidence that academic partnerships have produced graduates and faculty, generated curricula, and yielded research outputs. We can document with somewhat less confidence that countries with strong FM training systems have stronger primary care orientations than those without. We cannot, in most cases, attribute population-level outcomes to particular partnerships, because the causal pathways are too long, the confounders too numerous, and the counterfactuals unobserved.

A few cases come closer to causal claims. The Brazilian Family Health Strategy literature, including Macinko et al. (68) NEJM paper and the difference-in-differences and synthetic control analyses of Mais Médicos, has documented associations between primary care expansion and reductions in amenable mortality, hospitalizations for ambulatory care-sensitive conditions, and cardiovascular outcomes (41–43). The Flinkenflögel et al. (67) sub-Saharan scoping review documented graduate retention rates in family medicine programs that varied widely across countries, with some programs (notably South Africa, Botswana, and Uganda) demonstrating substantial retention in district hospitals and primary care. The Kruk et al. (80) Lancet Global Health Commission on High-Quality Health Systems in the SDG Era estimated that low-quality care, not lack of access, was responsible for more than five million amenable deaths annually in LMICs, an argument that puts educational quality at the center of the workforce conversation rather than at its periphery. The Hanson et al. (6) Lancet Commission on Financing Primary Health Care reinforced that workforce investments unaccompanied by financing reform are insufficient.

The most defensible position is that academic partnerships are necessary conditions for sustainable primary care workforce systems, that some partnerships contribute measurably to those workforce outcomes, and that the next decade requires far better evaluation infrastructure to test partnership-attributable impact. We return to this point in Section 11.

Part of the difficulty is that the field has measured activities and structures more often than outcomes. Much of the published evidence reports what partnerships did, namely the curricula written, the faculty trained, the papers co-authored, rather than what changed as a result, and the instruments used to judge partnership quality have until recently been heterogeneous and rarely validated. A 2025 scoping review by Modlin et al. (81) identified seventeen distinct equity toolkits for international academic partnerships, organized across domains such as governance, partnership dynamics, ethical foundations, and capacity, but found almost no evidence that using any of them actually produced more equitable practice. The gap is therefore not an absence of frameworks but an absence of validated, outcome-oriented evaluation. Implementation science offers one route forward, because its established constructs, including acceptability, adoption, fidelity, penetration, and sustainability, give partnerships a shared vocabulary for assessing not only whether an educational innovation works in principle but whether it is taken up and maintained in practice. Recent work applying the Consolidated Framework for Implementation Research to virtual educational capacity-building across Kenya, Guyana, and Turkiye illustrates how such frameworks can diagnose the barriers that determine whether a partnership’s outputs endure once external support recedes (82). We suggest that future partnership evaluation should pair equity appraisal with implementation outcomes and, wherever possible, with the kind of quasi-experimental designs already used in the Mais Medicos literature, so that the field can move from documenting effort to demonstrating effect.

It also matters that partnerships do not operate in a single environment, and the same modality can behave very differently depending on context. In well-resourced settings with mature academic infrastructure, the binding constraint on a partnership is rarely money or faculty but alignment of incentives and the avoidance of duplication. In many LMICs the constraints are more elemental, namely a thin specialist faculty base, dependence on external financing, and competition with service-delivery demands that crowd out protected academic time. In fragile and conflict-affected settings the calculus changes again, as continuity of personnel and physical security become preconditions that more stable partnerships can take for granted, and even well-designed collaborations can be interrupted overnight. South-South collaborations face a distinct configuration in which contextual fit and cultural authority are high but research financing and indexed-publication infrastructure are often weak, which is precisely why the transformative work described earlier has been undervalued in conventional metrics. A partnership model that succeeds in one of these environments may be inappropriate or even harmful in another, and the value of the typology lies partly in making these contextual contingencies visible rather than treating academic partnership as a single, uniformly beneficial intervention. Transformative rather than transactional partnership models, built on reflexivity, solidarity, and shared responsibility for outcomes, appear better suited to navigating this heterogeneity than the standardized templates that donor reporting tends to favor (83).

## 10 Challenges and the post-Astana agenda

The post-Astana decade has surfaced challenges that the academic partnership community must confront. Brain drain is the oldest and most familiar. The migration of family physicians and primary care nurses from LMICs to high-income countries continues to undermine the workforce gains that academic partnerships help to produce. Akey et al. (31) framing of bidirectional IMG pathways, alongside the WHO Global Code of Practice on the International Recruitment of Health Personnel, suggests that the response must be reciprocal investment rather than restriction.

Financing asymmetries persist. The Hanson et al. (6) Lancet Commission documented that PHC remains under-financed in most LMICs, that out-of-pocket payments continue to drive impoverishment, and that donor financing is often misaligned with country priorities. The financing emergency triggered by the 2025 retreat of several high-income countries from global health cooperation has sharpened these tensions, and the Lancet Primary Care editorial response has been unusually direct about the political economy of these decisions (45). Cultural adaptation has been more often invoked than studied. The Carvalho et al. (24) and Mencía-Ripley et al. (25) reflections offer entry points. What is missing is systematic comparative work on how partnerships adapt curricula and competency frameworks across cultural contexts.

The COVID-19 disruption forced rapid digital adoption that has, in many cases, been retained beyond the pandemic. The Sodhi et al. (53) paper on COVID-19 vaccination and PC-PH integration, the PAHO digital transformation work, and the multiple ECHO-style adaptations across Africa and Asia demonstrate that the digital pivot has expanded reach (76). It has also exposed inequities in connectivity and digital literacy that pre-existed the pandemic.

Climate-resilient primary care pedagogy is no longer a fringe concern. The Mash and Lokotola 2025 work, the Lokotola climate-resilient primary care framing, the Irlam, Scheerens, and Mash work on planetary health in African health professions education, and the AMEE consensus statement together signal that the next decade’s curricula must integrate planetary health as a core rather than elective domain (36, 63, 77, 78). The Lancet Commission on Planetary Health and the Mash et al. (78) climate vulnerability assessment in the Cederberg subdistrict in South Africa illustrate how partnership-based research can produce both evidence and curriculum content simultaneously.

Leadership for PHC reform is another live frontier. The Khalid et al. (44) Lancet Primary Care comment on the WHO Strengthening PHC Leadership Global Capacity Building Course, launched by WHO Academy in autumn 2024 with academic partners, articulates a model in which leadership is treated as a learnable, distributed competency rather than a charismatic attribute of senior officials. The course’s twelve-week blended structure, combining asynchronous content with live cross-country exchanges, is a contemporary illustration of how multilateral platforms and academic partners can co-deliver transformation. The Khalid et al. (44) parallel analysis of how thirty major international development organizations operationalize PHC documented persistent narrowing of the concept to service delivery, against the broader Astana vision. Academic partnerships have a role in resisting this narrowing through curriculum, research, and advocacy (84). The 2024 to 2027 Lancet Global Health Commission on People-Centered Care and the 2025 Lancet Americas Commission on PHC and Resilience together establish the policy-research scaffolding for the next phase (7–9).

The fragmentation of declarations is a quiet challenge. Astana, the Operational Framework, the Bogota-adjacent commitments around the 2022 Global Symposium on Health Systems Research in Bogota, the Alliance for PHC in the Americas, the Global Coalition of Countries on PHC, and multiple Lancet Commissions are not always coordinated, and ministries can experience the cumulative weight as advocacy fatigue rather than catalysis (5, 9, 45). Academic partnerships have a role in producing synthesis that helps countries navigate this density.

## 11 A research agenda for the next decade

A focused research agenda, mapped to SDG 3 and SDG 17 indicators, should anchor the next decade. Seven priorities arise directly from the gaps surfaced in this review, and Table 4 sets them out alongside their suggested study designs.

**Table 4:** Research agenda priorities mapped to SDG 3 and SDG 17 indicators.

Priority	Rationale	SDG indicator	Suggested study design
1. Causal partnership-workforce retention evidence	Move beyond association to causal attribution	SDG 3.c.1 health worker density	Synthetic control; staggered-rollout difference-in-differences
2. Expanded South-South evaluation literature	Counter publication asymmetry; build internal capacity	SDG 17.6 South-South cooperation	Multi-site mixed methods; networked cohort designs
3. Standardized equitable-authorship metrics	Operationalize SDG 17 partnership equity	SDG 17.16 multi-stakeholder partnerships	Bibliometric cohort with author-led co-design
4. Long-term sustainability beyond donor cycles	Five- to fifteen-year follow-up data scarcity	SDG 3.8 universal health coverage	Longitudinal program evaluation; retrospective cohort
5. Under-represented regions	Address Pacific Islands, Central Asia, Eastern Europe, francophone and Lusophone Africa gaps	SDG 3.c, 17.16	Regional consortium studies; multi-language scoping reviews
6. Indigenous-led partnerships and economic evaluation	Self-determination and cost-effectiveness frontier	SDG 10.2; SDG 3.8	Community-based participatory research; cost-effectiveness analysis
7. Planetary health integration and triangular partnership theory	Climate resilience and structural innovation	SDG 13.3 climate education; SDG 17.9 capacity building	Curriculum implementation studies; comparative case studies

The most urgent need is causal evidence of partnership-attributable workforce retention. Most of what we currently have is associational. Quasi-experimental designs that exploit staggered partnership rollouts, instrumental variables based on funding cycles, and synthetic control methods of the kind used in the Mais Médicos literature could be applied directly to academic partnership outcomes, and the field would gain considerably from such work (41, 42).

A second priority sits alongside the first: an expanded South-South evaluation literature. The PRIMAFAMED, CIMF, AfriWon, and South Asian networks should be supported to build evaluation infrastructure that produces internally driven, externally credible evidence of program performance, rather than waiting for Northern partners to ask the questions (17–19, 39, 40). The third priority is closely related, namely a small set of standardized equitable-authorship metrics that journals and funders adopt as routine reporting requirements; Beste et al. (59) nine themes and the decolonial partnership critique between them sketch the shape such indicators should take (25).

Sustainability is its own priority. Most evaluations end when funding ends, which means we know almost nothing about partnership outcomes at 5, 10, or 15 years. Long-horizon follow-up studies should be funded as a deliberate research investment rather than treated as an afterthought to project closeout reports.

Geography also matters. Several regions are systematically under-represented in the indexed academic partnership literature, including the Pacific Islands, Central Asia, Eastern Europe, francophone West Africa, and Lusophone Africa, and dedicated research investment in those regions would correct a long-standing distortion in the evidence base (24, 69). Indigenous-led partnerships and economic evaluation form a closely related sixth priority. The Lancet Americas Commission’s engagement with Indigenous representatives, the limited but growing Canadian and Australian work on Indigenous self-determination in curriculum design, and the broader gap in cost-effectiveness evidence on academic partnerships together represent intersecting frontiers that the next decade should not leave unstudied (9).

The final priority is theoretical as much as empirical: planetary health integration and the theory of triangular partnerships. The Mash, Lokotola, and PRIMAFAMED planetary health work, alongside the South African twinning experience and the Mais Médicos triangular model, point toward a generative agenda on how non-bilateral partnership structures perform under climate and political stress (34, 36, 41, 77). This is the kind of question that will only get sharper over the coming decade.

## 12 Conclusion

Seven years after Astana, academic partnerships in family medicine and Primary Health Care are visibly more numerous, more networked, and more reflexive than they were a decade ago. The Besrour Centre, PRIMAFAMED, the Iberoamerican Confederation, AfriWon Renaissance, the South Asian primary care community, the Lancet Commissions, the WHO Special Programme on PHC, and the Network: TUFH together constitute an emerging global academic infrastructure that did not exist in 1978 or even in 2008. The AP-PHC Typology we have proposed, with its five modalities mapped to Starfield’s 4Cs and the three WHO PHC components, offers a working scaffold for thinking about how this infrastructure performs and where its weaknesses lie.

The intellectual signature of this review is a falsifiable proposition. We want to be explicit that this is a hypothesis generated by the review rather than a finding established within it; the evidence we have summarized is largely descriptive and observational, and it can motivate but cannot yet confirm the claim. We assert that where academic family medicine partnerships explicitly embed the Besrour ten ethical principles and demonstrate equitable LMIC-led authorship, program sustainability beyond donor cycles is significantly more likely than where these conditions are absent. We mean this as a testable claim, not as a slogan. The post-Astana research community should design the comparative cohort studies, bibliometric analyses, and economic evaluations needed to subject this proposition to disconfirmation. If it proves false, we should revise it. If it proves true, the policy implications for funders, journals, and ministries are substantial.

The next decade will be hard. Financing emergencies and climate disruption will collide with demographic transition and political polarization, and every partnership the global academic community has built will be tested. Academic family medicine and Primary Health Care are uniquely positioned to respond, because their disciplinary commitments to first-contact access, longitudinality, comprehensiveness, and coordination, and their ethical commitments to integrated services, multisectoral action, and community empowerment, are precisely what the moment requires. We invite the global academic FM community to act on this responsibility with the seriousness it demands, to govern partnerships by explicit ethical principles rather than implicit habits, and to treat South-South leadership as a corrective presumption rather than the exception. This is meant to correct a long-standing default of Northern agenda-setting rather than to install a new hierarchy, and it carries an obligation to attend to those who can be marginalised even within Southern settings, including Indigenous communities whose self-determination must remain central rather than incidental. The world after Astana will not be transformed by declarations. It will be transformed, in classrooms and clinics from Vellore to Stellenbosch to Brasília to Kampala, by the people the academic community helps to form.
