# Supplementary material for: Academic partnerships for family medicine and primary health care education: a global narrative review 7 years after Astana
Source: Front Med (Lausanne). 2026 Jul 30;13:1870375. doi: 10.3389/fmed.2026.1870375 (PMC13467902; doi:10.3389/fmed.2026.1870375)
Supplement: Supplementary file 1 [file Table_1.DOCX]

**Supplementary File 1. SANRA Reporting Checklist**

Manuscript title: Academic Partnerships for Family Medicine and Primary Health Care Education: A Global Narrative Review Seven Years After Astana

Manuscript type: Review (narrative review)

Reporting standard: Scale for the Assessment of Narrative Review Articles (SANRA), Baethge C, Goldbeck-Wood S, Mertens S. Research Integrity and Peer Review. 2019;4:5. doi:10.1186/s41073-019-0064-8.

Scoring: each item rated 0 (low standard), 1 (intermediate), or 2 (high standard). Maximum total score 12.

**Summary of self-assessment**

| **Item** | **SANRA topic** | **Score** | **Reported on** |
| --- | --- | --- | --- |
| 1 | Justification of the article's importance for the readership | 2 | Section 1 (Introduction); Abstract |
| 2 | Statement of concrete aims or formulation of questions | 2 | Section 1 (final paragraph); Section 11; Section 12 |
| 3 | Description of the literature search | 2 | Section 2 (Methods) |
| 4 | Referencing | 2 | References 1 to 84; in-text citations throughout |
| 5 | Scientific reasoning, including appropriate presentation of data | 2 | Section 4; Tables 1 to 3; Figure 1; Sections 5 to 10 |
| 6 | Appropriate presentation of relevant endpoints | 1 | Section 9; Section 11 |
|  | Total self-assessed score | 11 / 12 |  |

**Item-by-item assessment with comments**

**Item 1. Justification of the article's importance for the readership**

**Score:** 2

**Reported on:** Section 1 (Introduction), paragraphs 1 to 5; Abstract.

***Comment.*** The Introduction situates the review against the 2018 Declaration of Astana, the 2020 WHO-UNICEF Operational Framework, the 2022 Lancet Global Health Commission on Financing PHC, the 2024 to 2027 Lancet Global Health Commission on People-Centered Care, and the 2024 to 2025 PAHO-World Bank-Lancet Regional Health Americas Commission, and identifies a specific gap: no recent narrative synthesis maps the full field of academic partnerships for family medicine and primary health care education to Starfield's 4Cs and the WHO three pillars, proposes a coherent typology, integrates the equity and decolonization critique structurally, and frames a falsifiable proposition for the next decade. Audience and policy relevance are stated explicitly, with academic partnerships framed as core implementation infrastructure for SDG 3, SDG 4, SDG 10, and SDG 17.

**Item 2. Statement of concrete aims or formulation of questions**

**Score:** 2

**Reported on:** Section 1 (final paragraph); Section 11 (Research Agenda); Section 12 (Conclusion).

***Comment.*** The Introduction closes with a defined aim and scope. The Conclusion advances a deliberately falsifiable proposition, namely that academic family medicine partnerships embedding the Besrour ten ethical principles and demonstrating equitable LMIC-led authorship will sustain beyond donor cycles more reliably than partnerships that do not. The seven-priority research agenda translates the synthesis into testable questions for the field.

**Item 3. Description of the literature search**

**Score:** 2

**Reported on:** Section 2 (Methods: Narrative Review Approach), paragraphs 2 and 3.

***Comment.*** The Methods section describes the databases queried (PubMed, Semantic Scholar, Google Scholar, ERIC), the journals hand-searched, citation tracking, search-term domains, the temporal scope of 2010 to April 2026 with seminal earlier works retained, the rationale for the 2010 cut point (publication of the Frenk Commission), and inclusion and exclusion logic. A dedicated paragraph also sets out how sources were screened, how partnership examples were selected (documentation quality, illustrative value, and geographic and structural diversity), and how the evidence was synthesised thematically against Starfield's 4Cs and the WHO three pillars. The choice of narrative rather than systematic methodology is explained and justified rather than left implicit.

**Item 4. Referencing**

**Score:** 2

**Reported on:** References 1 to 84 (Vancouver style); in-text citations throughout body.

***Comment.*** 84 numbered references in Vancouver format, as required by Frontiers. Every factual claim in the body is supported by at least one citation, every reference is cited at least once in the text, and every in-text citation number maps to an entry in the reference list. The reference set combines foundational sources (Starfield 2005, Macinko 2003, Frenk 2010), policy documents (WHO and UNICEF 2018 and 2020, PAHO 2023, multiple Lancet Commissions), and recent empirical and conceptual work, including 2025 sources on partnership equity toolkits, implementation science, and transformative research partnerships, with deliberate weighting toward LMIC-led scholarship. The 2026 McLellan and colleagues paper from the same Frontiers Research Topic is co-cited as a cognate contribution.

**Item 5. Scientific reasoning, including appropriate presentation of data**

**Score:** 2

**Reported on:** Section 4 (Typology); Tables 1 to 3; Figure 1; Sections 5 to 10.

***Comment.*** The manuscript builds an explicit conceptual frame, the AP-PHC Typology, derived inductively from the case literature and mapped deductively to two pre-specified frameworks (Starfield's 4Cs and the WHO three pillars). Each of the five typology modalities is illustrated with named exemplars and mapped to its distinctive contribution. Table 1 sets out the typology. Table 2 catalogs thirty-seven empirical academic partnerships. Table 3 maps the Besrour ten ethical principles to the WHO Operational Framework levers with worked examples. Figure 1 places the typology within the wider conceptual frame. Sections 5 through 10 walk through the evidence for each modality and theme with attention to limitations, contested claims, and donor-framing effects. Where evidence is thin or contested, this is stated explicitly.

**Item 6. Appropriate presentation of relevant endpoints**

**Score:** 1

**Reported on:** Section 9 (Workforce and Population Health Impact); Section 11 (Research Agenda).

***Comment.*** The strongest published evidence on PHC endpoints (Starfield Shi Macinko 2005 Milbank synthesis, the Brazilian Family Health Strategy literature, the Mais Medicos quasi-experimental and synthetic-control analyses, and the Kruk and colleagues 2018 Lancet Global Health Commission on High-Quality Health Systems) is presented in Section 9 with appropriate caveats about associational versus causal inference. Section 9 also now discusses evaluation frameworks and implementation science explicitly, noting that the field has measured activities more often than outcomes and that validated, outcome-oriented evaluation remains scarce. The manuscript is explicit that academic partnerships are necessary but not sufficient conditions for sustainable PHC workforce systems, that some partnerships contribute measurably to those workforce outcomes, and that the next decade requires far better evaluation infrastructure to test partnership-attributable impact. We have scored this item 1 rather than 2 deliberately, because the underlying literature on academic-partnership endpoints is itself uneven: most published evaluations end when project funding ends, and very few studies follow partnership outcomes at five, ten, or fifteen years. This is treated honestly as Priority 4 of the research agenda (long-term sustainability data beyond donor cycles) rather than papering over it.

**Total self-assessed SANRA score**

11 of 12. The score is offered in the spirit of methodological transparency. Final scoring properly belongs to peer reviewers and editors, and the authors welcome feedback on items where reviewer judgement differs from this self-assessment.

**Reflexivity and conflicts of interest**

The authors approached the literature with the prior conviction that family medicine and primary care are necessary, though not sufficient, conditions for equitable health systems, and that ethically governed academic partnerships are a legitimate vehicle for advancing them. A deliberate effort was made to foreground LMIC-led scholarship and to read Northern partnership accounts alongside the reflexive Southern critique rather than as the unmarked default. To preserve the global balance of the review and to avoid even the appearance of conflict of interest, the authors deliberately excluded examples, case studies, and partnerships involving the authors' shared affiliated institution, and recused themselves from citing their own published work on local programs. No external funding was received for this work and the authors declare no commercial or financial conflicts of interest.

**Reporting standard reference**

Baethge C, Goldbeck-Wood S, Mertens S. SANRA: a scale for the quality assessment of narrative review articles. Research Integrity and Peer Review. 2019;4:5. doi:10.1186/s41073-019-0064-8.

*Cited in the main manuscript as Reference 28.*
